# First report and genome sequencing of SARS-CoV-2 in a cat (*Felis catus*) in Colombia

**DOI:** 10.1590/0074-02760210375

**Published:** 2022-05-09

**Authors:** Yesica Botero, Juan David Ramírez, Héctor Serrano-Coll, Ader Aleman, Nathalia Ballesteros, Caty Martinez, Marina Muñoz, Alfonso Calderon, Luz H Patiño, Camilo Guzman, Sergio Castañeda, Yonairo Hererra, Salim Mattar

**Affiliations:** 1 Universidad de Córdoba, Instituto de Investigaciones Biológicas del Trópico, Montería, Colombia; 2 Universidad del Rosario, Facultad de Ciencias Naturales, Centro de Investigaciones en Microbiología y Biotecnología-UR, Bogotá, Colombia; 3 Universidad CES, Instituto Colombiano de Medicina Tropical, Medellín, Colombia

**Keywords:** population surveillance, public health, one health, COVID-19, viral zoonoses

## Abstract

**BACKGROUND:**

Severe acute respiratory syndrome coronavirus 2 (SARS-CoV-2) is a virus of zoonotic origin that can bind to ACE2 receptors on the cells of many wild and domestic mammals. Studies have shown that the virus can circulate among animals mutate, lead to animal-to-human zoonotic jump, and further onward spread between humans. Infection in pets is unusual, and there are few human-to-pet transmission reports worldwide.

**OBJECTIVE:**

To describe the SARS-CoV-2 infection in a domestic animal in Córdoba, Colombian Caribbean region.

**METHODS:**

A cross-sectional molecular surveillance study was carried out, oral and rectal swabs were taken from cats and dogs living with people diagnosed with coronavirus disease 2019 (COVID-19).

**RESULTS:**

SARS-CoV-2 was found in a cat living with a person with COVID-19. Genome sequencing showed that the B.1.111 lineage caused the infection in the cat. The owner’s sample could not be sequenced. The lineage is predominant in Colombia, and this variant is characterised by the presence of the D614D and Q57H mutation.

**CONCLUSION:**

The present work is the first report of an infected cat with SARS-CoV-2 with whole-genome sequencing in Colombia. It highlights the importance of detecting SARS-CoV-2 mutations that could promote the transmissibility of this new coronavirus. There is still a significant information gap on human-to-cat-to-human infection; we encourage self-isolation measures between COVID-19 patients and companion animals. The findings of this study give a preliminary view of the current panorama of SARS-CoV-2 infection in animals in Colombia.

Severe acute respiratory syndrome coronavirus 2 (SARS-CoV-2) appears to be of zoonotic origin;^1^ this new coronavirus can bind to ACE2 receptors in the cells of a wide variety of mammals.^1^ ^,^ ^2^ The infection has been described in humans and animals such as cats, dogs, ferrets, hyenas, coatis, otters, big cats (lions, tigers, panthers, pumas, leopards), non-human primates, white-tailed deer, manatees, hippopotamuses, hamsters, and minks naturally infected.^3^ ^,^ ^4^ ^,^ ^5^ To date, less than 400 cases describing animals infected with SARS-CoV-2 have been reported,^3^ a few with genomic analyses,^3^ ^,^ ^6^ ^,^ ^7^ and only one described in Colombia, a country located in the tropics that has been heavily hit by the pandemic.^8^

There are reports of humans that have transmitted the SARS-CoV-2 infection to animals. So far, only felines, rhesus macaque, mustelids, and Syrian hamsters have shown infection between individuals of the same species. The transmission of the virus from humans to big and small felines is evident, but the virus’s return to humans has not yet been demonstrated.^6^ ^,^ ^7^

The role that animals play in the dynamics of this infection is still unknown. However, some studies have shown that minks (*Neovison vison*) develop coronavirus disease 2019 (COVID-19), severe respiratory symptoms and death.^9^ Researchers have described that the virus has managed to infect humans again, introducing mutations in its protein S such as Y453F, F486L, and N501T, which could be of interest since they are directly involved in the binding and internalisation of the virus in host cells.^9^ ^,^ ^10^ ^,^ ^11^ Another study showed that the virus could circulate within hamsters and lead to human infections, as they described humans and hamsters infected with the Delta variant (AY.127).^4^ It strongly suggests that some pets can potentially be a secondary reservoir of SARS-CoV-2.^4^

The “One Health” approach offers a better understanding of these human-animal-environment interactions and enables better planning and decision-making around the public health of this emerging disease. Therefore, this research aimed to describe the SARS-CoV-2 infection in a domestic animal in Córdoba, Colombian Caribbean. The present study contributes information and awareness of the current panorama of SARS-CoV-2 infection in cats in the country.

## SUBJECTS AND METHODS

*Ethics approval and consent to participate* - The study follows the ethical standards of the Ministry of Health of Colombia Resolution No. 8430 of 1993. The present study data corresponds to one patient coded under strict anonymity with an internal laboratory number. This study is the results of a research project that was approved by the Ethics Committee of the Institute of Biological Research of the Tropic of the University of Córdoba, with the number No. 0410-2020 and by the Ethics Committee of the Faculty of Veterinary Medicine Act No. 005 26 (May 2021). All people involved in the research approved the publication. 

In a cross-sectional surveillance study, two hundred ninety samples were collected from domestic animals proportionally in eight municipalities of the Córdoba department located in the north-western Colombian Caribbean region. Informed consent was obtained from the pet’s legal guardians for sample collection in this study. Oropharyngeal and rectal swabs were collected for felines and canines as a part of the genomic and epidemiologic surveillance of pets living with COVID-19 patients’ households. Samples were conserved in a viral transport medium (VTM) for SARS-CoV-2 detection by reverse transcription real-time polymerase chain reaction (RT-qPCR).

A SARS-CoV-2 positive person who had signs and symptoms of COVID-19, like abdominal and chest pain, headache, and fever, alerted researchers from his cat, who died and was close to him. The cat died and showed respiratory manifestations 12 hours before. The animal was necropsied using the biosafety elements described for this procedure in animals suspected of SARS-CoV-2.^12^ ^,^ ^13^ ^,^ ^14^ According to the Organización Mundial de Sanidad Animal (OIE) and the Centers for Disease Control and Prevention (CDC), oral and rectal swabs were taken from other animals living in the same household and near the deceased cat’s home within a radius of approximately 500 metres.^13^ ^,^ ^14^

A surgical scalpel was used, a sample of different tissues (trachea, tonsil, and lungs) from the cat was macerated and homogenised within the viral transport medium. RNA extraction was performed in a BSL-2 laboratory with 200 µL of supernatant from each sample with the commercial GeneJET RNA Purification Kit™ (Thermo Scientific) based on lysis technology and silica-based membrane affinity columns following the manufacturer’s instructions. RNA was used to detect SARS-CoV-2 by amplifying the envelope gene (E gene) using the Biorad CFX96™ RT-qPCR system and C1000 Touch™ thermocycler, following the protocol’s primers and probes concentration recommendation published by Charité Hospital, Universitätsmedizin Berlin, Germany of Charité Berlin.^15^ Using Primer E_Sarbeco_F (400 nm per reaction), E_Sarbeco_R2 (400 nm per reaction), Probe E_Sarbeco_P1 (200 nm per reaction), and 1-Step RT-qPCR Kit Plus ROX Vial (Thermo Scientific™) to prepare the master mix. For each reaction, 15 µL of the master mix and 5 µL of controls were added to this mixture. RT-qPCR cycling conditions were one cycle (50ºC for 15 min), one cycle (95ºC for 15 min) and then 45 cycles (95ºC for 15 s), finally one cycle (60ºC for 1 min). The sample was considered positive with a Cq ≤ 35. The gene library was prepared from the RNA extracted from the trachea, and the ARTIC network protocol (https://artic.network/ncov-2019) was used. Long-read Oxford Nanopore MinION sequencing was carried out using the MinKNOW application (v1.5.5). The raw Fast5 files were named base and demultiplexed using Guppy. Then, the reads were filtered, eliminating the possible chimeric reads. Finally, the genome assemblies were obtained following the MinION pipeline described in the ARTIC bioinformatics pipeline (https://artic.network/ncov-2019/ncov2019-bioinformatics-sop.html). All assemblies were made based on the PANGOLIN nomenclature lineage mapper.^16^

## RESULTS

The cat owner was diagnosed by RT-qPCR (Cq 27.06) as COVID-19 with mild clinical manifestations. The cat was necropsied because of the diagnosis and samples were taken for RT-qPCR detection of SARS-CoV-2. Samples of the trachea (Cq 23.13), tonsil (Cq 32.59), and fecal swab (Cq 33.5) were positive to SARS-CoV-2 (Table I, Figure). The animal was neutered and vaccinated against rabies and lived with a 1-year-old dog and a 5-year-old cat. Both pets had outdoor access. The cat showed a history of scarring from fights with other domestic and feral animals. Samples were taken from the domestic animals living in the same household. Near the housing case, eleven cats and seven dogs’ samples were also taken; all of them were negative to the RT-qPCR test for SARS-CoV-2 (Table II).

**TABLE I:** Reverse transcription real-time polymerase chain reaction (RT-qPCR) results of the samples from the cat and its owner

Sample owner (D/M/Y)	Result RT-qPCR (Cq)
NPS (26/01/21)	Positive (27,06)
NPS (29/01/21)	Negative (38,14)
Sample cat (D/M/A)	Result RT-qPCR (Cq)
Trachea (26/01/21)	Positive (23,13)
Tonsil (26/01/21)	Positive (32,59)
Fecal swab (26/01/21)	Positive (33,5)
Lung (26/01/21)	Negative (N/A)

NPS: nasopharyngeal swab.

**Figure:**
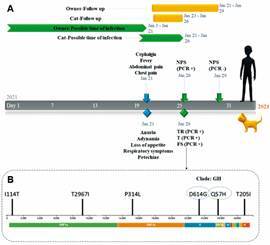
(A) Timeline of the diagnostic tests and onset of symptoms of the cat and its owner. The possible moment of contagion is speculative. NPS: nasopharyngeal swab; TR: trachea; T: tonsil; FS: fecal swab. (B) Severe acute respiratory syndrome coronavirus 2 (SARS-CoV-2) transcriptome.

**TABLE II:** Characteristics of domestic animals sampled that were in contact with the severe acute respiratory syndrome coronavirus 2 (SARS-CoV-2) positive cat

Variables	N = 18 (100%)
Species	
Feline	11 (61.1)
Canine	7 (38.9)
Positivity RT-qPCR	0

RT-qPCR: reverse transcription real-time polymerase chain reaction.

The tracheal purified RNA sample from the positive cat was sequenced, obtaining a genome sequence of the beta-coronavirus SARS-CoV-2 of the B.1.111 lineage (V. 2021-05-27), which presented mutations of interest previously described in Spike (D614G) and ORF3a (Q57H) and others that have not yet been described [Figure (B), Table III]. The owner’s sample could not be sequenced.

**TABLE III:** Whole-genome sequencing results

Sequence	hCoV-19/cat/Colombia/COR-U04/2021|EPI_ISL_2339859.2
Clade	20A (GH)
Total mutations	13
Substitutions	C241T, C346T, T606C, C1060T, C3037T, C9165T, C10507T, T13180G, C14408T, C18877T, A23403G, G25563T, C28887T

Most of the 290 domestic animals evaluated in the present work were cats. RT-qPCR and successful genome sequencing detected SARS-CoV-2 in a cat (EPI_ISL2339859.2) and a dog (EPI_ISL8422346) in two different cities for a total active infection rate of 0.69% in the department of Córdoba, Colombia (Table IV).^8^

**TABLE IV:** 

Sample size description and active infection rate	N = 290 (100%)
Species	
Cat	254 (87,6)
Dog	36 (12,4)
City	
Sahagún	29 (10)
Montelíbano	13 (4,5)
Lorica	25 (8,6)
Tierralta	23 (7,9)
Planeta Rica Montería	39 (13,4) 97 (33,4)
San Antero	20 (6,9)
Cereté	44 (15,2)
Positive RT-qPCR/NGS	2 (0,69)

NGS: next-generation sequencing; RT-qPCR: reverse transcription real-time polymerase chain reaction.

## DISCUSSION

A genome sequence of SARS-CoV-2 was obtained as reported by Ramírez et al.^16^ from the trachea sample belonging to the B.1.111 lineage (V. 2021-05-27). Genome analysis showed previously described mutations of interest in Spike (D614G) and ORF3a (Q57H) (Figure). The sequence obtained was placed in the GISAID virtual platform (EPI_ISL_2339859.2) (Figure). The genomic analysis for SARS-CoV-2 showed that it belongs to the phylogenetic Nextstrain clade 20A, and its lineages are currently circulating in Colombia. The lineage in which this sequence was classified is the predominant in the department of Córdoba (Colombia) and was detected for the first time in March 2020 with the D614G substitution in protein Spike, which gives the virus greater transmissibility, infectivity and increases the viral load in the respiratory tract.^17^ This mutation promotes an open conformation of the receptor binding domain (RBD), which facilitates the interaction of the SARS-CoV-2 Spike protein with ACE2 receptors.^16^ Therefore, it could also facilitate better interaction and affinity of the SARS-CoV-2 RBD with the ACE2 receptors of domestic animals such as dogs or cats.

Another significant mutation is Q57H, which is a poorly studied mutation that could be related to the greater transmissibility of SARS-CoV-2. This mutation occurs on the ORF3a protein, a viporin capable of acting with an ion channel, thus promoting the release of viral genetic material in the host cell.^18^ Therefore, Q57H would further stabilise the quaternary structure of this viporin, facilitating the improved release of viral particles by promoting pore formation in the host cell.^19^ Therefore, this mutation should be studied in greater depth in the pathogenesis of SARS-CoV-2 in both humans and animals.

On the other hand, Renata et al.^6^ implemented a genomic analysis to suggest that the transmission of SARS-CoV-2 from a person to their pet is likely. The feline infected with SARS-CoV-2 was found in Montería, Córdoba. Montería is the capital of the department where the study was carried out. According to the Instituto Nacional de Salud of Colombia,^20^ it has a higher population density of pets and humans and a more significant number of reported COVID-19 in humans (51% cases and 47% deaths of the department).

Another study reported high seropositivity for COVID-19 in people from the department of Córdoba (40.8%).^21^ Altogether a high density of pets and a high density of people with COVID-19 could increase the risk of transmission of SARS-CoV-2 to domestic animals.

Also, it should be noted that the population of domestic cats and dogs in the Colombian Caribbean region tends to street lifestyle, increasing the contact of pets with other animals and people, which increases the risk of transmission of this virus to other species. However, in this study, the animals in contact with the positive cat had negative results.

Finally, this is the first report of SARS-CoV-2 genome sequencing in a cat in Colombia. Relevant data that contributes to the understanding of the role played by domestic animals in the dynamics of infection was obtained. To date, some studies of natural and experimental infection in animals have shown that dogs, felines, primates, and farm animals such as minks have a greater susceptibility to this betacoronavirus.^22^ However, birds such as chickens, ducks, geese, and quail, which have been associated with the transmission of multiple respiratory viruses, one of them the avian influenza virus A(H5N1), A(H7N9), do not appear to be susceptible and they act as intermediate hosts for SARS-CoV-2.^22^ ^,^ ^23^ Still, the appearance of new mutations in the RBD segment of protein S could promote a greater susceptibility of this coronavirus to a more significant number of animals, promoting the appearance of new variants of SARS-CoV-2.

Therefore, it is still necessary to carry out other studies under the “One Health” approach, including detailed knowledge of the viral circulation in humans and animals^24^ - using other methods such as serological tests to obtain more evidence about SARS-CoV-2 prevalence in animals.

Although there are few human-to-pet transmission reports worldwide, SARS-CoV-2 has a wide host range.^3^ ^,^ ^4^ ^,^ ^5^ Therefore, we encourage self-isolation measures between COVID-19 patients and companion animals to prevent the appearance of zoonotic events that could lead to new mutations in the virus.

In conclusion, the findings of this study give a preliminary view of the current panorama of SARS-CoV-2 infection in animals in Colombia. The work contributes to a better understanding of the animal-human interface in the epidemiology of this disease.
